# Gait analysis related to functional outcome in patients operated for ankle fractures

**DOI:** 10.1002/jor.24071

**Published:** 2019-04-12

**Authors:** Sander van Hoeve, Michael Houben, Jan P. A. M. Verbruggen, Paul Willems, Kenneth Meijer, Martijn Poeze

**Affiliations:** ^1^ Division of Trauma Surgery, Department of Surgery Maastricht University Medical Center P. Debyelaan 25, PO Box 5800 Maastricht 6202 AZ The Netherlands; ^2^ Department of Movement Sciences Maastricht University Medical Center P. Debyelaan 25, PO Box 616 Maastricht 6200 MD The Netherlands; ^3^ School for Nutrition and Translational Research in Metabolism NUTRIM PO Box 616 Maastricht 6200 MD The Netherlands

## Abstract

Ankle fractures are among the most common lower limb fractures. Associations between postoperative radiographic results and clinical outcome have been found, but less is known about the relevant ankle biomechanics. This study analyzed ankle kinematics, radiographic findings, and patient‐reported outcome measures (PROM) in patients treated for ankle fractures. The hypothesis was that patients after ankle fracture surgery had less flexion/extension in the ankle compared to healthy subjects and that fracture severity had significant influence on kinematics and patient satisfaction. Thirty‐three patients (*n* = 33 feet) operated for ankle fractures were recruited. Ankle kinematics were analyzed using the Oxford Foot model, and results were compared with an age‐matched healthy control group (11 patients, 20 feet). In addition, patients were divided by fracture (severity) classification and kinematic results were correlated with PROM and radiographic findings. Patients treated for ankle fracture showed lower walking speed (*p* < 0.001) when asked to walk in preferred normal speed. When compared at equal speed, significantly less range of motion (ROM) between the hindfoot and tibia in the sagittal plane (flexion/extension) during loading and push‐off phases (*p* = 0.003 and *p *< 0.001) was found in patients after ankle fractures compared to healthy subjects. Lowest ROM and poorest PROM results were found for patients with trimalleolar ankle fractures. There was a significant correlation between ROM (flexion/extension) during the push‐off phase and SF‐36 physical functioning (*r* ^2^ = 0.403, *p* = 0.027) and SF‐36 general health (*r* ^2^ = 0.473, *p* = 0.008). Fracture severity was significantly correlated with flexion/extension ROM in the ankle during both loading and push‐off phases (*r* ^2^ = −0.382, *p* = 0.005, and *r* ^2^ = −0.568, *p* < 0.001) and was also significantly correlated with PROM. This study found that patients with ankle fractures had significantly altered ankle kinematics compared to healthy subjects. The poorest results were found among patients with trimalleolar fractures. Weak to strong significant correlations were found between fracture severity, ankle kinematics, and PROM. © 2019 The Authors. *Journal of Orthopaedic Research*® Published by Wiley Periodicals, Inc. on behalf of Orthopaedic Research Society. J Orthop Res 37:1658–1666, 2019.

Ankle fractures are among the most common lower limb fractures and account for 9% of all fractures.^1^, ^2^, ^3^, ^4^ There are various classification systems to describe ankle fractures, for example, the AO‐classification, the Lauge–Hanssen classification, and the number of fractured malleoli.^5^, ^6^, ^7^, ^8^, ^9^, ^10^ Functional outcome studies have been performed among patients with ankle fractures, analyzing patient‐reported outcome measures (PROM), physical examination, and radiographic findings.^11^, ^12^, ^13^ These studies reported good results, with patients experiencing little or mild pain and few restrictions in functional activities 1 year after ankle fracture surgery.^11^, ^12^, ^13^, ^14^, ^15^, ^16^ Several studies examined the association between the severity of the fracture and the functional outcome, with mixed results. Some concluded that classification of the fracture can be used as a predictor of functional outcome after surgery.^17^, ^18^ Others, like Egol et al.,^19^ concluded that the type of fracture had no influence on functional outcome after ankle fracture surgery. Kinematic analysis may provide a more robust predicator of patient‐reported functional outcome, and may provide a better correlation between fracture severity and patient satisfaction. Only a few studies have analyzed gait in patients treated for ankle fracture.^9^, ^20^, ^21^, ^22^ These studies found reduced flexion/extension in patients treated for ankle fracture compared to healthy subjects. However, no or minimal significant correlations were found between gait and PROM. Kinematic analysis of the foot and ankle can be performed with reliable results using a multi‐segmented foot model such as the Oxford foot model (OFM).^23^, ^24^

In the present study, a kinematic analysis was performed in patients operated for ankle fractures, and the results were compared with those of healthy subjects. Furthermore, kinematic results were compared between patients based on fracture (severity) classification, the number of fractured malleoli. In addition, kinematic results were correlated with PROM and radiographic findings. The hypothesis for this study was that after operative treatment for ankle fracture, patients would have reduced flexion/extension of the ankle compared to healthy subjects, and that this would be significantly correlated with PROM and fracture (severity) classification. The poorest results were expected in patients operated for trimalleolar ankle fractures.

## METHODS

### Study Population

This study was performed between 2010 and 2014 at the Maastricht Universitary Medical Centre. For this prospective, single‐center, level II evidence study 33 patients (33 feet) were recruited prospectively after operative treatment for an unstable ankle fracture. All patients were diagnosed with a malleolar ankle fracture between 2010 and 2013 at the emergency department. Diagnosis was formed on anteroposterior, mortise, and lateral radiographs. If necessary, a computed tomography scan was performed. All patients were operated by an experienced foot and ankle surgeon within 2 weeks after injury. Fixation was performed with plate and screw osteosyntheses following the AO‐principles.^25^, ^26^ For all patients immobilization with cast for 6 weeks was perscribed and permissive weight‐bearing started after these 6 weeks, supported with physiotherapy. Patients had at least 6 months follow‐up (range 7–57) and were included at least 6 months after the operation, as most functional progress after surgical intervention takes place between 3 and 6 months.^19^, ^27^, ^28^ Patients were contacted for this study during follow‐up initially at the outpatient clinic by the researcher. If willing to participate patients were contacted every 3 months by the researcher, separately from normal follow‐up at the outpatient clinic. Patients were included for this study when they had reached good mobility in the foot and ankle after surgery. This was when patients had returned to work, had no physiotherapy and stopped using painkillers. Subsequently, patients were planned for gait analysis within 4 weeks. Patients were included average 18 months (range 7–57) after surgery. Patients with an age range between 25 and 75 years were included, since age has limited effect on ankle kinematics.^29^, ^30^, ^31^ Exclusion criteria were contralateral or ipsilateral fractures or pre‐existent abnormalities of the lower extremities, neurotrauma, spinal, or neurological injury, and pathologic fractures. 26% of all patients operated for ankle fractures between 2010 and 2013 at our institution, who met the inclusion criteria, were included in the study. Results were compared with a group of age‐matched healthy subjects (*n* = 11). Subjects were recruited around the university of Maastricht. Since previous gait studies found asymmetric gait patterns in healthy subjects with differences in foot dynamics between the dominant and non‐dominant leg, also kinematics may alter between both legs in healthy subjects.^32^, ^33^, ^34^, ^35^ To correct for foot dominance in all healthy subjects both feet were analyzed. Foot dominance was tested, asking patients, and healthy subjects to step on an elevation and to kick a soccer ball with one leg. The leg they used was found to be their dominant leg.^34^ All patients and healthy subject had a right dominant leg.

### Recruitment, Foot Dominance, Operatie En Post Op Traject

#### Equipment

At the movement laboratory, motion stereophotogrammetry was used with the OFM. Motion capture was conducted using the VICON MX 3‐system (Vicon Motion Systems Ltd., Oxford, UK). The VICON system comprises eight cameras (6 MX3 and 2 T20, running at 200 Hz) for the detection of markers placed on the skin of the lower extremity. The markers were placed on specific anatomic points on the subjects using regular double‐sided tape, according to the guidelines for the OFM.^23^, ^31^, ^36^ A 10‐m platform was used for patients and healthy subjects to walk on, with the force platform (Kistler 9282E) in the middle. Vicon Nexus was used to visualize and process the 3D motions on the computer.

#### Study Protocol

Each subject was analyzed individually at the movement laboratory, where he or she underwent all measurements on the same day. The following data was collected for the patients operated after ankle fracture: baseline characteristics including height, weight, body‐mass index (BMI; weight/height^2^), leg length and knee and ankle width, and American Society of Anaesthesiologists physical status classification system (ASA‐classification).^37^ In addition, PROM were collected. The Foot and Ankle Disability Index [FADI] consists of 26 items with five answers from no difficulty at all to unable to do. The FADI score ranges from 0 to 100 with a score of 100 indicating an excellent or maximum outcome. The Short‐Form 36 score [SF‐36] consists of 36 questions, and measures functional health and well being, dived in eight domains: physical functioning, physical role, pain, general health, vitality, social functioning, emotional role, and mental health. The score for each domain ranges from 0 to 100. A higher score means a better outcome. The visual analogue pain scale [VAS] for maximum, minimum, and current pain ranges from 0 to 10 with 0 in patients with no pain and 10 in patients with unsustainable pain. The American Orthopaedic Foot and Ankle Society hindfoot‐ankle score [AOFAS] consists of seven items (pain, activity limitations, footwear, walking distance, walking surface, gait abnormalities, and alignment) and ranges from 0 to 100 points, with 100 points indicating an excellent or maximum outcome. Questionnaire were filled out by the researcher prior to the gait analysis by asking patients the questions in Dutch. Physical examination of the lower extremities was performed by the researcher prior to gait analysis.^38^, ^39^ Using plain pre‐operative radiographs and computed tomography scans, fractures were classified by the number of fractured malleoli and by the AO‐classification.^5^, ^6^, ^7^, ^8^, ^9^ This was performed by two observers blinded for other results in this study. The kinematic analysis was performed following the OFM protocol described in detail in our previous studies.^23^, ^31^, ^36^ Patients and healthy subjects were asked to walk in self‐selected normal, slow, and fast speed. All measurements were performed by one researcher (S. van Hoeve), who had been trained in foot examination and OFM. The data of one whole step was divided into two intervals: a loading phase and a push‐off phase. Walking speed was compared between groups. Further, intersegment ROM between hindfoot and tibia (location of the talocrural joint) in the frontal, sagittal, and transverse planes (representing abduction/adduction, flexion/extension, and inversion/eversion, respectively) were calculated.^40^, ^41^ The kinematic results of the patients operated for ankle fracture were compared with those of healthy subjects. Since speed has significantly influence on kinematics, kinematics will be compared between groups with equal speed during walking.^42^ In addition, kinematic and PROM results were compared between patients with unimalleolar, bimalleolar, and trimalleolar fractures. The kinematic results were correlated with fracture (severity) classification and PROM. All subjects signed and gave informed consent. The medical ethics committee of this hospital approved this study.

#### Data Analysis

Before patients were included, a power analysis was performed, using a power and sample size calculator, to determine the number of patients needed for inclusion. The calculator used two averages (α = 5% and 1‐β = 80%). The value of ROM between the hindfoot and tibia (talocrural joint) in the sagittal plane (flexion/extension) in healthy subjects during gait was known to be 12.0° (SD 3.0).^23^ After ankle trauma surgery, the ROM in patients was expected to have decreased by at least 1 standard deviation, or 3°, leading to a ROM of 9°. This resulted in a number of subjects to be included of at least eight for each group.^43^ Data was converted using Matlab (version 7.12,2011) and analyzed in SPSS (IBM Statistics, version 20). The data comprised the mean of at least six randomly chosen trials (steps). The Shapiro–Wilk test was used to test the distribution of data and found a normal distribution. The patients characteristics and categorial variables were analyzed using descriptive statistics and the independent samples *t*‐test was used to find significant differences, a *p*‐value below 0.05 being considered statistically significant. Speed between groups was compared with the independent samples *t*‐test. The Pearson correlation test was used to identify significant correlations between kinematics and PROM. For the categorical variables the Spearman rank correlation test was used to find significant correlations between number of fractured malleoli and AO‐classification with PROM and kinematics.

## RESULTS

### Patient Characteristics

The demographic characteristics and PROM for the patients operated for ankle fractures and the healthy subjects are presented in Table 1. Thirty‐three patients were included in the ankle fracture group and 11 healthy subjects were included in the healthy control group. Due to a technical error during gait analysis in one healthy subject only one feet was analyzed. Therefore, in the healthy group 20 feet were included in this study, with 10 dominant and 10 non‐dominant feet. The mean age of the patients in the ankle fracture group was 57.2 ± 14.5 years (range 25–78), while that of the healthy control group was 48.4 ± 16.0 years (24–65). The ankle fracture group comprised significantly more women with a lower height and a lower leg length compared to the healthy subject group. No further significant differences were found between the two groups. Based on the number of fractured malleoli, 10 patients were included in the unimalleolar group, 11 in the bimalleolar group, and 12 in the trimalleolar group. Using the AO‐classification, one patient was classified as having an A2 fracture, 17 patients as having a B2 ankle fracture, nine as having a B3 fracture, two as having a C1 fracture, and four as having a C2 fracture. The mean FADI score was 87.2 ± 14 (SD) (range 35–100), while the mean VAS pain score during walking was 1.8 ± 2.2 (SD) (range 0–7) and the mean AOFAS hindfoot‐ankle score was 84.2 ± 14.9 (SD) (range 44.2–100). Gait measurements were performed at a mean of 18 ± 9 (range 7–57) months after surgery.

**Table 1:** Patient Characteristics and Patient‐Reported Outcome Measures in Patients Treated for Ankle Fracture and Healthy Control Subjects

	Ankle Fractures	Healthy Control	*p*‐Value
Patient (*n* and no. of feet)	33, 33	11, 20	
Age (years)	57.2 ± 14.5 (25–78)	48.4 ± 16.0 (24–65)	0.079
Gender (*n*, % male)	18, 54.5%	9, 81.8%	**0.025**
Side (*n*, % right)	19, 57.6 %	10, 50.0 %	0.600
Dominant foot (*n*, %)	19, 57.6 %	10, 50.0%	0.600
Height (m)	1.73 ± 0.1 (1.56–1.93)	1.80 ± 0.05 (1.69–1.85)	**0.008**
Weight (kg)	79.9 ± 14.7 (54–115)	78.0 ± 10.0 (63–91)	0.686
BMI	26.6 ± 4.2 (20.5–36.1)	24.2 ± 3.0 (19.4–29.1)	0.090
Knee width (mm)	102.5 ± 12.3 (80–138)	104.5 ± 6.6 (93–114)	0.507
Ankle width (mm)	71.8 ± 5.3 (58–85)	69.9 ± 5.0 (62–77)	0.209
Leg length (mm)	891.7 ± 58.1 (750–1000)	914.5 ± 21.3 (900–970)	**<0.001**
Patient‐reported outcome measures			
FADI	87.2 ± 14 (35–100),		
VAS current pain	1.8 ± 2.2 (0–7)		
SF‐36			
Physical functioning	75.1 ± 25.0 (25–100)		
Pain	61.2 ± 28.3 (10−100)		
Vitality	68.3 ± 22.3 (30−100)		
General health	70.3 ± 17.5 (35−95)		
Physical role	44.2 ± 43.4 (0−100)		
Emotional role	68.9 ± 42.8 (0−100)		
Social functioning	65.0 ± 28.9 (12.5−100)		
Mental health	74.5 ± 19.3 (36−100)		
AOFAS ankle‐hindfoot score	84.2 ± 14.9 (44.2−100)		

Results are presented as mean ± standard deviation and (minimum–maximum).

Significant different values are indicated in bold.

### Kinematic Analysis

Table 2 lists the kinematic results for the patients operated for ankle fracture and for the healthy subjects. A significant difference in walking speed between the two groups was found when patients were asked to walk at preferred normal speed (*p* < 0.001). This significance disappeared when healthy subjects were asked to walk slowly and the ankle fracture patients walked at normal speed (*p* = 0.360). When adjusted for speed, the ROM between the hindfoot and tibia in the sagittal plane (flexion/extension) during both the loading and push‐off phases was lower in the ankle group than among the healthy subjects (*p* = 0.003 and *p* < 0.001, respectively). There were no significant differences between the two groups in the ROM in the frontal plane (abduction/adduction) and transverse plane (inversion/eversion), neither during the loading phase nor during the push‐off phase.

**Table 2:** Walking Speed and Ankle KinematicsSignificant different values are indicated in bold

	Groups			*p*‐Value	
Variables	Ankle Fracture Group (33,33)	Healthy Subjects (11,20)		Ankle gr. vs. Healthy Subjects	
Patient (*n* and No. of Feet)	Normal Speed	Normal Speed	Slow Speed	Normal vs. Normal	Normal vs. Slow
Speed (m/s)	0.88 ± 0.23 (0.43 −1.35)	1.24 ± 0.19 (0.91 −1.59)	0.94 ± 0.20 (0.54 −1.23)	**<0.001**	0.360

	Ankle Group	Healthy Subjects	
	Normal Speed	Slow Speed	Ankle vs. Healthy Subjects
Hindfoot‐tibia loading phase			
Sagittal plane	7.90 ± 2.25	10.13 ± 2.98	**0.003**
Flexion/extension	(3.15−11.97)	(3.86−14.78)	
Frontal plane	11.35 ± 4.97	11.93 ± 3.23	0.645
Abduction/adduction	(4.09−30.04)	(7.22−18.09)	
Transverse plane	5.85 ± 1.89	6.43 ± 2.10	0.308
Inversion/eversion	(1.87−10.46)	(2.95−11.21)	
Hindfoot‐tibia push‐off phase			
Sagittal plane	8.41 ± 3.06	12.59 ± 3.73	**<0.001**
Flexion/extension	(3.60−15.93)	(5.32−18.35)	
Frontal plane	12.91 ± 5.31	10.60 ± 4.66	0.116
Abduction/adduction	(4.63−25.49)	(5.84−27.25)	
Transverse plane	8.68 ± 3.33	10.63 ± 3.65	0.051
Inversion/eversion	(1.97−14.96)	(4.94−17.89)	

Results are presented in degree as mean ± standard deviation and (minimum–maximum).

Significant different values are indicated in bold.

### Kinematic Analysis of Patients Classified by Number of Fractured Malleoli

**Figure 1:**
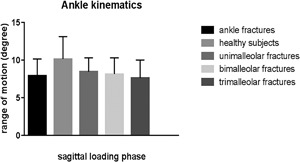
Range of motion in the sagittal plane during the loading phase for patients operated for ankle fractures (unimalleor, bimalleolar, and trimalleolar) and healthy subjects.

**Figure 2:**
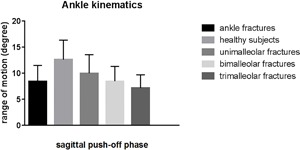
Range of motion in the sagittal plane during the push‐off phase for patients operated for ankle fractures (unimalleor, bimalleolar, and trimalleolar) and healthy subjects.

Table 3 lists the characteristics of the patients with unimalleolar, bimalleolar, and trimalleolar ankle fractures. There were no significant differences between the groups regarding age, gender, or fracture side. Fracture mechanism, ASA‐classification, time to surgery, physiotherapy, and complications were recorded. Postoperatively, one patient with a unimalleolar fracture developed a deep infection, which was treated with antibiotics and removal of the osteosynthesis material. There were no patients with open fractures. Table 4 presents the kinematic and PROM results. There was a significantly lower ROM between hindfoot and tibia in the sagittal plane (flexion/extension) during the push‐off phase in the patients with a trimalleolar ankle fracture compared to patients with a unimalleolar fracture (*p* = 0.042). The FADI was significantly lower in the trimalleolar group compared to the bimalleolar group (*p* = 0.007). The AOFAS score was significantly lower in the trimalleolar group compared to the unimalleolar and bimalleolar groups (*p* = 0.032 and *p* = 0.006, respectively). Figures 1–2 shows the flexion/extension ROM values of the healthy subjects and the patients with ankle fractures, as well as the results for unimalleolar, bimalleolar, and trimalleolar ankle fractures.

**Table 3:** Patient Characteristics Classified by Number of Fractured Malleoli

	Groups			*p*‐Value		
Malleoli	Unimalleolar (1)	Bimalleolar (2)	Trimalleolar (3)	1 vs. 2	1 vs. 3	2vs. 3
Patients (*n* and no. of feet)	10, 10	11, 11	12, 12			
Age (years)	51.5 ± 10.7 (38–73)	56.1 ± 14.9 (25–70)	62.9 ± 15.9 (32–78)	0.432	0.068	0.302
Gender (*n*, % Male)	7, 70%	7, 63.6%	4, 33.3%	0.772	0.095	0.160
Side (*n*,% right)	5, 50 %	6, 54.5 %	8, 66.7 %	0.845	0.453	0.573
Fracture mechanism	9 fall 1 traffic accident	11 fall	9 fall 1 traffic accident			
ASA‐classification	7 ASA 1	7 ASA 1	5 ASA 1			
	3 ASA 2	4 ASA 2	6 ASA 2			
			1 ASA 3			
Complications	1 infection	0	0			
Physiotherapy	7 patients average 8 months (1–15)	7 patients average 4 months (1.5–6)	11 patients average 9 months (2–24)			
Time to surgery	Average 6 days (0–17) 5 delayed 5 within 1 day	Average 3 days (0–12) 4 delayed 7 within 1 day	Average 6 days (0–21) 5 delayed 7 within 1 day			
Tertius fixation	−	−	7 fixed 5 not fixed			

Results are presented as mean ± standard deviation (minimum–maximum).

**Table 4:** Kinematics and Patient‐Reported Outcome Measures for Patients With Unimalleolar, Bimalleolar, and Trimalleolar Ankle Fractures

Variables	Groups			*p*‐Value		
Group	Unimalleolar	Bimalleolar	Trimalleolar	Uni vs. Bi	Uni vs. Tri	Bi vs. Tri
Patient (*n* and no. of feet)	(10, 10)	(11, 11)	(12, 12)			
Speed (m/s)	1.03 ± 0.14 (0.75−1.23)	0.94 ± 0.17 (0.67−1.13)	0.88 ± 0.19 (0.53−1.12)	0.241	0.051	0.383
Hindfoot‐tibia loading phase						
Sagittal plane	8.44 ± 1.85	8.13 ± 2.16	7.59 ± 2.42	0.724	0.373	0.584
Flexion/extension	(5.14−11.13)	(5.59−11.97)	(3.81−10.84)			
Frontal plane	12.33 ± 2.99	12.32 ± 6.64	11.97 ± 6.57	0.996	0.867	0.900
Abduction/adduction	(6.71−15.95)	(5.23−30.04)	(3.76−22.69)			
Transverse plane	6.18 ± 2.15	5.81 ± 1.51	6.01 ± 2.83	0.650	0.873	0.841
Inversion/eversion	(2.80−10.10)	(3.80−8.62)	(2.47−11.36)			
Hindfoot‐tibia push‐off phase						
Sagittal plane	9.97 ± 3.57	8.45 ± 2.84	7.13 ± 2.55	0.292	**0.042**	0.253
Flexion/extension	(5.81−15.93)	(3.60−12.25)	(3.03−11.16)			
Frontal plane	12.65 ± 3.04	14.35 ± 5.06	12.80 ± 7.25	0.359	0.952	0.562
Abduction/adduction	(8.28−17.12)	(7.74−21.60)	(4.53−26.28)			
Transverse plane	10.27 ± 2.86	8.95 ± 2.95	7.52 ± 3.73	0.313	0.071	0.321
Inversion/eversion	(5.93−14.50)	(3.36−14.96)	(2.53−13.40)			
Patient‐reported outcome measures						
FADI	86.3 ± 7.7 (76.0−98,10)	93.3 ± 9.5 (71.2−100)	75.2 ± 17.7 (44.2−100)	0.114	0.074	**0.007**
AOFAS	91.7 ± 7.3 (82.0−100.0)	94.8 ± 5.9 (84.0−100.0)	77.5 ± 17.1 (35.0−100.0)	0.315	**0.032**	**0.006**

Results are presented as mean ± standard deviation and (minimum–maximum).

Significant different values are indicated in bold.

### Correlations

There were significant correlations between the ROM in the sagittal plane during the push‐off phase (flexion/extension) and the VAS pain score (*r* ^2^ = −0.382, *p* = 0.044), the SF‐36 physical functioning score (*r* ^2^ = 0.403, *p* = 0.027), the SF‐36 social functioning score (*r* ^2^ = 0.495, *p* = 0.005), the SF‐36 physical role score (*r* ^2^ = 0.378, *p* = 0.039), the SF‐36 vitality score (*r* ^2^ = 0.419, *p* = 0.021), the SF‐36 general health score (*r* ^2^ = 0.473, *p* = 0.008), and the AOFAS ankle‐hindfoot score (*r* ^2^ = 0.414, *p* = 0.020). There was also a significant correlation between the ROM in the sagittal plane during the loading phase and the AOFAS ankle‐hindfoot score (*r* ^2^ = 0.363, *p* = 0.044) (Table 5). Fracture severity as classified by the number of fractured malleoli was significantly correlated with the flexion‐extension ROM between the hindfoot and tibia during both the loading and push‐off phases (*r* ^2^ = −0.382, *p* = 0.005, and *r* ^2^ = −0.568, *p* < 0.001, respectively). The number of fractured malleoli was also significantly correlated with the SF‐36 physical functioning score (*r* ^2^ = −0.678, *p* < 0.001), the SF‐36 physical role score (*r* ^2^ = −0.370, *p* = 0.044), the SF‐36 emotional role score (*r* ^2^ = −0.445, *p* = 0.014), the SF‐36 mental health score (*r* ^2^ = −0.631, *p* < 0.001), the SF‐36 vitality score (*r* ^2^ = −0.689, *p* < 0.001), the SF‐36 pain score (*r* ^2^ = −0.362, *p* = 0.049), the SF‐36 general health score (*r* ^2^ = −0.512, *p* = 0.004), and the AOFAS ankle‐hindfoot score (*r* ^2^ = −0.414, *p* = 0.021). There was a significant correlation between the AO‐classification and the SF‐36 general health score (*r* ^2^ = −0.524, *p* = 0.004). The AO‐classification was also significantly correlated with the flexion/extension ROM during push‐off in the ankle (*r* ^2^ = −0.389, *p* = 0.028).

**Table 5:** Correlations Between Ankle Kinematics, Patient‐Reported Outcome Measures, and Fracture Severity

Motion	FADI	VAS	Physical Functioning	Social Functioning	Physical Role	Emotional Role	Mental Health	Vitality	Pain	General Health	AOFAS	Malleoli	AO‐Classification
Hindfoot‐tibia loading phase													
Flexion/extension	0.176	−0.131	0.102	0.150	0.110	0.079	−0.134	0.026	−0.103	0.231	0.363*	−0.382**	0.032
Abduction/adduction	0.056	0.082	0.097	0.037	−0.087	0.011	0.013	0.027	0.046	−0.126	−0.048	−0.056	−0.060
Inversion/eversion	0.102	−0.052	0.292	0.082	0.025	0.151	0.076	0.237	0.131	0.001	0.223	−0.130	‐0.219
Hindfoot‐tibia push‐off phase													
Flexion/extension	0.287	−0.382*	0.403*	0.495**	0.378*	0.330	0.344	0.419*	0.286	0.473**	0.414*	−0.568**	−0.389*
Abduction/adduction	0.396*	−0.380*	0.168	0.207	‐0.096	0.047	0.151	0.077	0.355	0.041	0.437*	0.194	‐0.020
Inversion/eversion	0.346	−0.405*	0.489*	0.426*	0.411*	0.238	0.587**	0.452*	0.544**	0.274	0.385*	−0.310*	−0.501**

Results are presented as correlations *(r)*, with **p value <0.01 and *p value <0.05 in bold.

## DISCUSSION

The present study evaluated ankle kinematics, PROM, and fracture severity in patients operated for ankle trauma. After an average of 18 months follow‐up, we found that patients operated for unstable ankle fractures had lower walking speed, when asked to walk at preferred normal speed, and less flexion/extension between hindfoot and tibia compared to healthy subjects, when walked at equal speed. The smallest flexion/extension and poorest PROM results were seen in patients with trimalleolar ankle fractures. The flexion/extension ROM during push‐off phase was weak to strong significantly correlated with PROM and the severity of the ankle fracture as defined by the number of fractured malleoli and the AO‐classification.

A number of previous studies have compared gait patterns among patients treated for ankle fractures with those of healthy subjects. Losch et al. analyzed gait in 20 patients with a surgically treated ankle fracture one year after operation, and compared the results with those of 20 healthy adults. They found less flexion/extension in the ankle joint and lower speed and smaller step length in the injured group compared to the healthy subjects. However, they did not find any significant correlation between kinematic parameters and PROM.^21^ Our study also found lower walking speed and less flexion/extension among patients operated for ankle fracture. We did, however, find a weak to strong significant correlation between fracture severity, PROM and ankle kinematics. Wang et al.^22^ analyzed 18 patients with an ankle fracture using PROM and gait, with a multi‐segment foot model (modified Oxford foot model) at least 1 year post‐operatively. Twelve patients had a lateral unimalleolar fracture and six had a trimalleolar fracture, and all were treated with open reduction and internal fixation. Results were compared with those of healthy subjects and the contralateral leg. The study found less flexion/extension between the hindfoot and tibia in the fracture group compared to the healthy subjects during stance, and lower ROM (flexion/extension) in the swing phase compared to the non‐injured side. They found that the Olerud and Molander ankle score (OMAS) questionnaire correlated fairly to moderately with the kinematic parameters in the sagittal plane during swinging phase (flexion/extension. Their results are comparable with those found in our study. Our gait study included a larger number of patients and found multiple weak to strong significant correlations between kinematics, fracture classification and PROM, adding new data to the present literature.

Two studies have analyzed plantar pressure, walking speed, and step length in patients treated for ankle fractures. Segal et al. analyzed functional outcome in 41 patients with ankle fractures using PROM, physical examination, and walking speed and step length. Patients with an ankle fracture were divided into those with a unimalleolar fracture (*n* = 12), those with a bimalleolar fracture(*n* = 15), and those with a trimalleolar fracture (*n* = 14). Gait results were compared with those of 72 healthy subjects. They found significant differences between the groups for all parameters, including walking speed and involved and uninvolved step length. Patients with a unimalleolar fracture performed significantly better than those with a bimalleolar or trimalleolar ankle fracture.^9^ Becker et al. analyzed 40 patients with displaced ankle fracture by physical examination, PROM and measurement of plantar pressure distribution. In their group, 32 patients had suffered a unimalleolar ankle fracture and eight a bimalleolar fracture. Results were compared with those of 90 healthy subjects. They found differences in loading pressures between patients with high and low PROM scores.^20^ These two studies both showed that patients with less severe fractures had better outcomes, which is in line with the findings of our study.

Other studies investigating functional outcome after ankle fractures reported mixed results with respect to the relationship between fracture severity and functional outcome. Broos et al.^17^ found that patients with unimalleolar fractures had a better outcome than those with trimalleolar fractures in terms of clinical and radiographic findings. Hancock et al.^18^ found that subjects with unimalleolar and bimalleolar ankle fractures had better functional outcome than those with trimalleolar fractures, based on the OMAS and the Lower Extremity Functional Scale (LEFS). In contrast, Egol et al.^19^ concluded that the type of fracture had no influence on functional outcome after ankle fracture surgery, with fractures categorized according to the Orthopaedic Trauma Association (OTA) system and the Lauge–Hansen system. Our study provides evidence for the hypothesis that the severity of ankle fractures correlates with functional outcome, as we found a weak to strong significant correlation between fracture severity (in terms of the number of fractured malleoli and the AO‐classification) and several PROM, with the SF‐36 physical functioning and AOFAS ankle‐hindfoot scores.

Our analysis of the results of our study found that ankle kinematics were decreased most in the patients with severe ankle fractures (trimalleolar). The AO‐classification also correlated significantly with the flexion/extension in the ankle joint during the push‐off phase. Since the patient characteristics did not differ significantly between the groups (unimalleolar, bimalleolar, and trimaleolar), the results of our study provide a reliable objective indication of differences between groups. We found decreasing flexion and extension in the ankle joint in patients with increasing severity of the fracture, although no significant differences in ROM were found between the patients with the unimalleolar and bimalleolar fractures. Comparison of the patients with trimalleolar ankle fractures with the healthy subjects showed a significantly lower ROM (7.13° ± 2.55° (SD) vs. 12.59° ± 3.73° (SD)). In line with this, the PROM scores were equal between the unimalleolar and bimalleolar groups, while the reported functional outcome was significantly lower in the trimalleolar group. This seems to indicate that a decrease in ROM of more than 4.5° in the push‐off phase is clinically significant.

A number of limitations can be listed for our study. There were significantly differences in patient characteristics between the ankle fracture group and the healthy subject group. In addition, patients after ankle fractures had significantly lower speed. When healthy subjects walked at slow speed no significant differences between both groups were found. For further analysis the most important characteristics regarding foot and ankle kinematics as speed and foot dominance did not significantly differ.^32^, ^33^, ^34^, ^35^, ^42^ The characteristics that differ between both groups had no significantly influence on foot and ankle kinematics. Therefore, the risk of bias of type 2 error is limited. In contrast to previous studies the group of healthy subjects was limited with 11 subjects and in this group both feet were analysed to rule out the influence of foot dominance.

Although the OFM, which we used, is the most commonly used foot model for clinical studies, it cannot directly measure the motion in the talocrural joint: it measures the ROM between the hindfoot and tibia segments. Markers are placed on the calcaneal bone for the hindfoot segment and on the malleoli of the tibia segment, so the model cannot measure the exact motion between talus and tibia, but can only provide an estimation. However, when we started this study, no multi‐segment foot model was available to measure the exact motion between talus and tibia. Marker placement can result in errors as well as skin motion. To reduce this error, the observer was trained in placing the markers. Another limitation was that no additional analysis was performed of the surgical reduction of the fracture. In patients with a trimalleolar fracture in particular, the residual gap or step‐off of the tertius fragment in the talocrural joint can influence motion. Hence, additional studies are warranted.

## CONCLUSION

This study found that at an average of 18 months after ankle surgery, patients had significantly lower walking speed when asked to walk at preferred normal speed and significantly lower ROM in the sagittal plane (flexion/extension) of the ankle compared to healthy subjects, compared at equal speed. The smallest flexion/extension and poorest PROM results were found for the patients with trimalleolar fractures. Weak to strong significant correlations were found between fracture severity (based on the number of fractured malleoli and the AO‐classification), the ROM in the sagittal plane and the PROM.

## AUTHORS’ CONTRIBUTIONS

Gait analysis and writing by SVH. Spss analysis and writing by MH. Operation and correction writing by JPAMV. Technical aspects of gait analysis and matlab analysis by PW. Writing by KM. Operation and writing by MP. All authors state they had an important contribution to this manuscript. All the Authors have read and approved the final submitted manuscript.
